# Temporal Summation of Pain Is Not Amplified in a Large Proportion of Fibromyalgia Patients

**DOI:** 10.1155/2012/938595

**Published:** 2012-06-03

**Authors:** Stéphane Potvin, Emilie Paul-Savoie, Mélanie Morin, Patricia Bourgault, Serge Marchand

**Affiliations:** ^1^Centre de Recherche Fernand-Seguin and Department of Psychiatry, Faculty of Medicine, Université de Montréal, QC, Canada H1N 3V2; ^2^Department of Surgery, Faculty of Medicine and Health Sciences, Université de Sherbrooke, Sherbrooke, QC, Canada J1H 5N4; ^3^School of Nursing, Faculty of Medicine and Health Sciences, Université de Sherbrooke, Sherbrooke, QC, Canada J1H 5N4

## Abstract

*Background*. Recently, it has been proposed that fibromyalgia (FM), a chronic widespread pain syndrome, results from overactive endogenous excitatory pain mechanisms. Experimental studies using temporal summation paradigms have confirmed this hypothesis but have included small samples of patients, prompting our group to perform a large-scale study. *Methods*. Seventy-two female FM patients and 39 healthy females participated in the study. The temporal summation test consisted of a 2-minute continuous and constant heat pulse administered with a thermode on the participants' left forearm. Experimental temperature was set at a value individually predetermined to induce a 50/100 pain rating. *Results*. Relative to controls, FM patients had lower thermal pain thresholds and lower temporal summation of pain. However, 37 FM patients required experimental temperatures lower than the minimal temperature used in controls (45°C). Nevertheless, temporal summation was not increased in the other FM subgroup, relative to controls, despite equivalent experimental temperatures. *Discussion*. Our results suggest that temporal summation of pain is normal, rather than increased, in a large proportion of FM patients. Future studies on temporal summation in FM will need to be careful since some FM patients require abnormally low experimental temperatures that may confound results and make necessary to separate patients into subgroups.

## 1. Introduction

Fibromyalgia (FM) is defined by widespread chronic musculoskeletal pain [1, 2], despite the absence of a clinically demonstrable nociceptive cause. Although FM leads to substantial social, economic, and health consequences, the pathophysiology of the disorder remains poorly characterized. In the last decade, the use of psychophysical and electrophysiological procedures has produced evidence suggesting an implication of the central nervous system in fibromyalgia. More precisely, it has been proposed that FM may result from overactive endogenous excitatory pain mechanisms [3].

 The temporal summation paradigm is the experimental model the most frequently used in humans to study endogenous excitatory pain mechanisms (e.g., central sensitization). Temporal summation results in an amplification of pain perception following repeated or continuous administration of constant noxious stimuli [4, 5]. Temporal summation of pain is thought to reflect the progressive enhancement of C-fiber-evoked responses of dorsal horn neurons (windup) and seems to be dependent on N-methyl-D-aspartate (NMDA) receptor mechanisms in both animals [6] and humans [7].

 In human experimental settings, temporal summation is usually elicited using phasic repetitive pain stimuli administered at short interstimulus intervals (≥0.3 Hz) [8–10] or continuous tonic stimuli [11–13]. Over the last decades, numerous studies using* repetitive* (thermal and mechanical) noxious stimuli have repeatedly shown that temporal summation effects are more pronounced in FM patients, relative to healthy controls [14–17], strongly suggesting that FM is an endogenous state of central sensitization. However, the great majority of studies have been performed thus far in small samples of FM patients (*n* ≈ 15). Although such small-scale studies may have sufficient sample size to examine differences between FM patients and healthy controls, they are not suited to determine the rate of FM patients who have exaggerated windup. FM is a highly heterogeneous disorder, whose symptoms (fatigue, pain, anxiety, depression, sleep problems, etc.) vary considerably from one patient to another, and the pathophysiological processes underlying these symptoms may also vary considerably across subgroups of FM patients [18]. In this vein, our group has gathered abundant evidence that FM is associated with substantial heterogeneity in psychophysical pain measures, such as heat pain thresholds, cold hyperalgesia, and pain inhibition efficacy [19–21]. Recently, our group developed a paradigm using continuous thermal noxious stimuli to measure temporal summation in humans [13]. Using this simple and easily tolerated paradigm, the current study sought to measure temporal summation of heat pain in a large sample of FM patients and to determine whether temporal summation is homogeneously increased in this population.

## 2. Methods

### 2.1. Participants

Seventy-two women suffering from FM and 39 healthy women participated in this study aged between 18 and 65 years old. Patients were diagnosed with FM using *American College of Rheumatology *criteria [1] by neurosurgeons, rheumatologists, or physicians specialized in chronic pain. That is, FM patients all suffered from diffuse pain lasting more than 3 months, and all had ≥11 tender points, as determined by the tender point assessment that was performed as part of the routine medical examination. None of the patients were referred by psychiatrists. Participants (patients and controls) who were pregnant or breastfeeding, who had diabetes, lupus, rheumatoid arthritis, or suffering from a cardiac pathology were excluded from the study. Patients and controls did not differ in terms of age and presence/absence of menstrual cycle (Table 1). None of the FM patients were treated with opioid analgesics. Twenty-seven FM patients received antidepressants and 17 received anticonvulsants at the moment of psychophysical testing. The Human Ethics Committees of the *Université du Québec en Abitibi-Témiscamingue* and *Université de Sherbrooke *approved the research protocol, and all participants gave their written, informed consent.

### 2.2. Clinical Assessment

The symptoms of FM were assessed using the French version of the self-administered *Fibromyalgia Impact Questionnaire* (FIQ) that measures the components of health (pain, stiffness, fatigue, anxiety, depression, physical functioning, work status, and well-being) most affected by FM over the last week [22]. The French version is widely used by researchers and clinicians and has acceptable internal consistency, test-retest reliability, and construct validity [23].

### 2.3. Experimental Pain Measures

#### 2.3.1. Thermal Pain Thresholds

In a pretest at the beginning of the session, thermal pain thresholds (TPTs) were measured by applying a thermode on the left forearm of participants. The Peltier thermode used (TSA II, Medoc, Advanced medical systems, Minneapolis, MN 55435) was a heating plate connected to a computer, which allowed a precise setting of experimental temperature. Subjects were advised that the thermode temperature would gradually increase from 32°C to 51°C (maximum) by a rate of 0.3°C per seconds until it reached their thermal pain tolerance. During the full thermal stimulation, pain intensity was continuously measured (at 1 Hz) using a computerized visual analog scale (COVAS), which ranged from 0 (no pain) to 100 (most intense pain tolerable). Subjects were also instructed to start displacing the COVAS' cursor when their sensations changed from heat to pain (TPTs). For each subject, the procedure was repeated twice to ensure the stability of measurement of the TPTs and the experimental temperature.

#### 2.3.2. Temporal Summation of Heat Pain

The temporal summation test was completed after the pretest and consisted of a continuous heat pulse administered with a thermode for 2 minutes on the left forearm of participants. Experimental temperature reached a predetermined fixed value and remained constant during the 2-minute testing period (Time 0 to Time 120). It was set at a value corresponding to a temperature individually predetermined to induce a 50/100 pain rating during the 2 pretesting sessions used to determine the individual's TPT. That is, during the pretests, the experimenter noted the temperature associated with a 50/100 pain rating, as displayed on the Medoc software (laptop), for each subject. For the temporal summation stimulation, participants were not told that the temperature remains constant throughout testing, after reaching the individualized experimental temperature (Time 0). During the thermal temporal summation stimulation, pain intensity was also measured using the COVAS. Research in our laboratory has shown that pain perception scores increase through the 2 minutes of testing, most prominently during the last 15 seconds, even if the thermode temperature remains constant [13, 24], suggesting a temporal summation effect. Importantly, pain ratings at Time 0 (when the experimental temperature is reached) were very close to 50/100 and did not differ between FM patients and healthy controls (see Table 1).

### 2.4. Statistical Analyses

For statistical purposes, we used 3 dependent variables, namely, (i) TPTs, (ii) experimental temperature, and (iii) temporal summation (Mean COVAS score_Time15–30 s_  minus Mean COVAS score_Time105–120 s_) [13]. Analyses of variance (ANOVAs) were conducted to explore the potential differences between FM patients and healthy volunteers. The critical level of significance for rejecting the null hypothesis was set at 5%.

## 3. Results

### 3.1. FM Patients versus Healthy Controls (HC)

**Figure 1:**
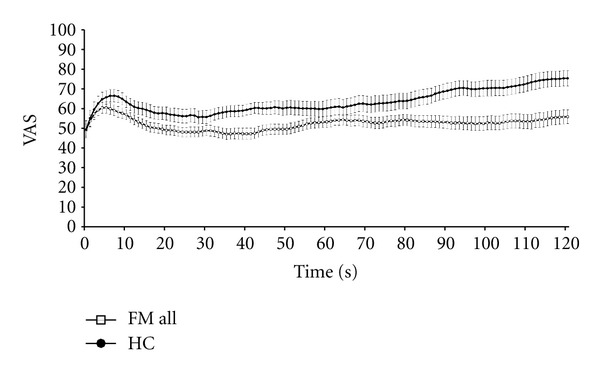
Temporal summation of heat pain fibromyalgia patients and healthy controls. This figure depicts the time course of pain perception during the tonic thermal noxious stimulation in fibromyalgia (FM) patients (open circles) and healthy controls (HCs) (black lines). We can clearly see that HCs have more pain at the end of the curve (temporal summation) when compared to FM patients; error bars refer to standard errors.

Relative to healthy controls, FM patients (as a whole) had lower TPTs and lower experimental temperature (Table 1). Temporal summation of pain was also decreased in FM patients, relative to controls, although this difference failed to achieve significance (Table 1, Figure 1). Noticeably, a positive Pearson's correlation was found between experimental temperature and the rate of temporal summation in FM (*r* = 0.331; *P* = 0.005). Finally, none of the pain measures significantly differed between FM patients with and without antidepressants, as well as FM patients with and without anticonvulsants (all *P* > 0.05).

### 3.2. FM Patients (≥ or <45°C) versus HC

**Figure 2:**
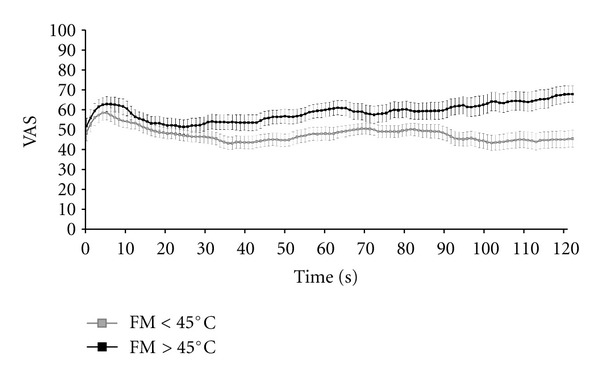
Temporal summation of heat pain in fibromyalgia subgroups. This figure depicts the time course of pain perception during the tonic thermal noxious stimulation in fibromyalgia (FM) patients receiving experimental temperatures lower and higher than 45°C. We can clearly see a temporal summation effect in the FM ≥ 45°C subgroup that was not present in the FM < 45°C subgroup; error bars refer to standard errors.

Given that FM patients required lower experimental temperatures to achieve 50/100 pain ratings, and that it has been shown that low-intensity nociceptive stimuli elicit less (or even no) temporal summation than high-intensity stimuli in healthy subjects [25], we performed secondary analyses to determine the influence of experimental temperature on our results. More precisely, we subdivided FM patients into those who received thermal stimulation lower and higher than 45°C, since 45°C was the lowest experimental temperature used in our sample of HC [Note: None of the controls required an experimental temperature lower than 45°C to induce a 50/100 pain rating, thus, none needed to be excluded on that basis]. Post hoc analyses revealed that FM patients ≥/<45°C and controls differed in terms of TPTs and temporal summation (Table 1, Figure 2). After applying Bonferoni correction, multiple comparisons revealed that FM ≥ 45°C patients did not differ from HC in terms of temporal summation of pain and TPTs. The FM < 45°C subgroup had lower TPTs and lower temporal summation of pain, relative to the 2 other groups. Importantly, the experimental temperature used during the 2-minute thermal noxious stimulation did not differ between FM ≥ 45°C patients and controls, but it was obviously decreased in FM < 45°C patients, relative to the 2 other groups (Table 1). Also noteworthy, the FIQ total score and the FIQ pain item did not differ between FM patients whose experimental temperature was higher and lower than 45°C (Table 1).

## 4. Discussion

As shown previously by our group and others, TPTs were lower in FM relative to HC [19, 21, 26, 27], suggesting that FM may not only be associated with mechanical hyperalgesia but also with thermal hyperalgesia. When examining temporal summation in a large group of FM patients, we found decreased temporal summation of pain in FM, relative to HC. However, the experimental temperatures used in the FM group as a whole were significantly lower than the temperatures used in controls, meaning that both groups did not receive noxious heat stimulations of similar intensities. To overcome this methodological limitation, FM patients were subdivided into those receiving thermal stimulations higher and lower than 45°C. Importantly, we found that the group receiving stimulations higher than 45°C did not have increased temporal summation of pain, relative to controls, despite equivalent thermal stimulation intensities. This latter result strongly suggests that a large proportion of FM patients (here, 35 out of 72) do not have overactive endogenous excitatory pain mechanisms, a result which is unequivocally inconsistent with repeated findings from other groups [14–17]. As such, this finding deserves greater consideration.

One possible explanation for the lack of increased or even lack of temporal summation of pain in FM is that we only included female patients in our study, whereas most previous studies on the topic included both male and female participants [14–17]. However, this factor is unlikely to explain the discrepancy between our results and previous ones, because increased temporal summation of pain has been shown in both male and female FM patients [14–17]. Another potential reason may lie in the fact that we employed a temporal summation paradigm using continuous heat stimuli instead of repetitive heat pulses, as used previously by other groups [14–17]. However, this factor is also unlikely to explain per se the discrepancy between our results and previous ones, because an experimental study by Granot and colleagues [28] directly compared both procedures in healthy subjects and found that phasic and tonic stimuli produce similar temporal summation effects. Still, by employing repetitive heat pulses of short durations (~1 sec), researchers are habitually able to use elevated thermal stimuli (range: 47–51°C) to induce temporal summation of pain in FM patients [14, 15, 17], something that was difficult to achieve here, in a subgroup of FM patients, by using tonic stimuli lasting 2 minutes. In a closely related matter, it must be considered that in our study, individualized experimental temperatures were used, whereas previous groups used fixed temperatures in most cases [14, 15, 17]. That is, all participants (FM patient and controls) received identical noxious stimuli in the previous fixed-temperature protocols, whereas FM patients received lower thermal stimulations, compared to controls, in our individualized-temperature protocol. Since FM patients had lower TPTs in our study, some FM patients expectedly required lower experimental temperatures to achieve 50/100 pain ratings. This may explain why FM patients (as a whole) had decreased (nonsignificant trend) temporal summation of pain, relative to controls. It may also explain why there was no temporal summation effect at all in the FM < 45°C subgroup. Indeed, the experimental temperatures that this subgroup of patients could tolerate were probably too low (e.g., 42.6°C) to induce significant thermal windup. As such, this observation shows that the use of individualized experimental temperatures may be problematic when studying thermal pain windup in *some* chronic pain patients who are particularly hypersensitive to thermal pain.

We acknowledge that these methodological differences (continuous stimuli, individualized temperatures) between our procedure and the procedures used previously by other labs may explain the discrepancy of the results obtained in the FM < 45°C patients. However, the critical issue here is that these differences cannot rule out the main finding of our study, namely, that a large subgroup of FM patients do not have increased temporal summation of heat pain. Despite the fact that the experimental temperatures used in the FM ≥ 45°C patients and the controls were nearly identical (46.2 versus 46.4°C, resp.), the FM ≥ 45°C subgroup did not display enhanced temporal summation of pain, relative to controls. That is, despite the use of sufficiently noxious stimuli (≥45°C) to induce temporal summation, pain amplification was normal in large proportion of FM patients (close to 50/100). This raises the possibility that previous studies were not able to demonstrate the existence of this large subgroup of FM patients because they had very small samples of patients, clearly underpowered to detect subgroup effects and not designed to do so. Moreover, using a fixed temperature rather than a temperature adjusted to pain perception may be problematic. For the same stimulation intensity, the perceived pain is significantly higher in FM patients. This augmented perception may be the sole explanation for the increased temporal summation. The augmentation of the healthy subjects' experimental temperatures to the perceived pain of FM patients may have further increased the level of temporal summation of heat pain observed in these subjects.

Intriguingly, both FM subgroups did not differ in FIQ total and FIQ pain scores. Although the exact meaning of this result remains elusive, it may suggest that thermal pain hypersensitivity, as displayed by the FM < 45°C subgroup, is not a pathognomonic feature of FM, since the FM ≥ 45°C patients had significant levels of FM symptoms without having significantly reduced TPTs, relative to HC. Apart from the well-described mechanical allodynia, other pain mechanisms could be altered in these FM patients, including deficient endogenous pain inhibition, autonomic dysfunctions, enhanced appraisal of pain, and/or abnormal inflammatory responses [29]. Further studies on the heterogeneity of altered pain mechanisms in FM are required.

Overall, our results strikingly cast some doubt on the whole idea that endogenous excitatory pain mechanisms are overactive in the great majority of FM patients. However, it is important to be cautious when interpreting our results, since we cannot rule out that temporal summation may have emerged as significantly increased in the FM < 45°C subgroup if we had used another temporal summation procedure. Indeed, pain amplification has been repeatedly reported to be more pronounced in FM patients, relative to healthy controls, in seminal studies performed by other groups using fixed experimental temperatures [14–17]. Moreover, our own study comprised a large subgroup of FM patients (*n* = 37) having very low TPTs, who all had experimental temperatures lower than 45°C, suggesting that this subgroup of FM patients was already sensitized to pain from the very start of thermal stimulations. Using fixed-temperature noxious stimuli to elicit temporal summation, it seems possible that we may have found this subgroup to have increased temporal summation of pain, relative to controls. If lower experimental temperatures are required to elicit 50/100 pain ratings in the FM < 45°C subgroup, it seems likely that fixed temperatures across groups would have been rated as more painful in the FM < 45°C patients, relative to controls. Head-to-head comparisons of temporal summation paradigms using repetitive versus continuous thermal stimuli as well as individualized and fixed temperatures will need to be performed in large samples of FM patients in order to verify this assumption.

Although the temporal summation results of the FM < 45°C subgroup must be taken cautiously for obvious methodological reasons, the lack of increased pain amplification in the subgroup of FM patients who received significant levels of tonic thermal noxious stimuli (≥45°C) has to be taken seriously. As such, this latter result may have decisive implications for the identification of FM subgroups and the characterization of underlying physiopathological processes. For instance, the finding of increased pain amplification in FM has prompted the hypothesis that glutamatergic disturbances may be involved in the physiopathology of FM [3]. The results of the current study suggest that this hypothesis will need to be examined in FM while bearing in mind that FM is not a homogeneous syndrome, both clinically and physiologically.

## Figures and Tables

**Table 1:** Clinical and psychophysical differences between fibromyalgia patients and healthy controls.

Variable	FM (*n* = 72)	<45°C (*n* = 37)	>45°C (*n* = 35)	HC (*n* = 39)	Statistics (2 groups)	Statistics (3 groups)
Age	47.6 (9.8)			44.8 (9.5)	*F* = 2.1; *P* = 0.147	

Menstrual cycle	Yes, 27; no, 45			Yes, 18; no, 18*	*χ* ^2^ = 2.7; *P* = 0.259	

Antidepressants		Yes, 12; No, 24*	Yes, 15; No, 20		*χ* ^2^ = 0.7; *P* = 0.409	

Anticonvulsants		Yes, 9; No, 25*	Yes, 8; No, 27		*χ* ^2^ = 0.1; *P* = 0.728	

FIQ total score		58.1 ± 15.3	56.0 ± 13.3		*F* = 0.4; *P* = 0.549	

FIQ pain item		7.1 ± 2.2	7.0 ± 1.7		*F* = 0.1; *P* = 0.795	

Pain rating at time 0 (%)	50.1 ± 24.2	48.6 ± 25.3	51.7 ± 23.4	50.4 ± 24.7	*F* = 0.003; *P* = 0.954	*F* = 0.1; *P* = 0.862

TPTs (°C)	39.9°C ± 3.7	38.6 ± 2.5	41.3 ± 4.1	42.2 ± 3.2	*F* = 10.9; *P* = 0.001	*F* = 12.1; *P* = 0.0001**

Experimental temperature (°C)	44.4°C ± 2.3	42.6 ± 1.7	46.2 ± 0.9	46.4°C ± 1.0	*F* = 27.3; *P* = 0.0001	*F* = 106.6; *P* = 0.0001**

Temporal summation (Δ)	4.8 ± 25.9	−2.9 ± 25.1	12.9 ± 24.7	14.9 ± 29.0	*F* = 3.5; *P* = 0.068	*F* = 5.0; *P* = 0.008**

Δ: delta, FIQ: Fibromyalgia Impact Questionnaire, FM: fibromyalgia, HC: healthy controls, TPTs: thermal pain thresholds, ± refers to standard deviations.

*missing data for a few subjects; **FM lower than 45°C < 2 other groups (Bonferroni correction).
